# The Physical Environment and the Quality of Life and Behavior in People With Dementia: A Systematic Meta-Review

**DOI:** 10.1177/30495334251345092

**Published:** 2025-06-13

**Authors:** Arnout Siegelaar, Mark P. Mobach, Sarah Janus, Sytse U. Zuidema

**Affiliations:** 1 University of Groningen, The Netherlands; 2 Hanze University of Applied Sciences Groningen/The Hague University of Applied Sciences, The Netherlands; 3 The Hague University of Applied Sciences, The Netherlands; 4 University Medical Center Groningen, The Netherlands

**Keywords:** behavior, dementia, Alzheimer, nursing home, physical environment, quality of life

## Abstract

The environmental design of care settings is increasingly recognized as an important factor that can support reducing neuropsychiatric symptoms and improving quality of life in people with dementia. This review provides a comprehensive overview of consensual knowledge of specifications of the environmental design that are found to be associated with quality of life and behavior, that includes evaluation of the strength of evidence. Seven databases were searched, yielding 410 unique reviews. Assessment of relevance to the subject and assessment of the evidence level of those specifications. The selection process yielded 11 systematic reviews. The results show that a broad range of architectural features are beneficiary to quality of life and behavior, but the level of evidence is low. There is a large body of consensual knowledge on environmental design that is associated with quality of life and behavior, but the quality of evidence is low. Finding a balance between overstimulation of senses and sensory deprivation is a challenge in designing environments for people with dementia. Providing variation in ambiance of spaces may have beneficiary effects on behavior and quality of life.

## Introduction

In 2019, 55.2 million people suffered from dementia worldwide, and the projected number in 2050 increases to 139 million. In 2019, an estimated 8,5 million people with dementia (PwD) were registered living in long-term care facilities (LTCF) worldwide, according to WHO (2021). The economic impact of dementia is considerable; the annual costs of dementia are over USD 1.3 trillion and are expected to rise to USD 2.8 trillion by 2030, of which 40% is spent on professional and residential care (Prince, 2015). In the Netherlands, the number of PwD expected to increase from 290,000 in 2022 to 620,000 in 2050 (Alzheimer Nederland, n.d.). A proportion of these people, currently estimated at close to 80,000, is admitted to a purpose-built LTCF. By September 2023, an additional 13,563 PwD waited to be admitted to a LTCF (Zorginstituut, n.d.).

Factors determining the need for purpose-built long-term care were researched in a European prospective cohort study (Verbeek et al., 2015) that identified three categories of factors associated with transition from living at home to a LTCF. PwD who had made a transfer to a LTCF had a lower cognitive status, displayed more severe depressive and other neuropsychiatric symptoms, and were more likely to live alone and to have an informal caregiver who experienced a higher caregiver burden than people who remained living at home. de Vugt et al. (2005), Verbeek et al. (2015), van Hoof et al. (2009), and Smith et al. (2022) suggest that the physical environment plays an important role in the adverse effects of this transition; these must also be understood in a wider context of factors, for instance, the shortage of trained staff, financial limitations, and the progressive nature of dementia.

Furthermore, the environmental design of care settings is increasingly recognized as an important factor that can support reducing neuropsychiatric symptoms and improving quality of life (QoL) in PwD. Although robust evidence of the impact of environmental design is said to be scarce (Harrison et al., 2022), environmental modifications may be an effective non-pharmacological factor in reducing neuropsychiatric symptoms (Soril et al., 2014). Activities of daily living (Reimer et al., 2004), social engagement, dementia related outcomes (Ferdous, 2020; Marquardt et al., 2014) and QoL in general (Reimer et al., 2004) may further improve purpose-built environments in special care units (SCUs), when compared to traditional residential care facilities. The design of the environment may even contribute to therapeutic goals (Abraha et al., 2017; Brawley, 2001; Calkins, 2009; Chaudhury et al., 2013; Werezak & Morgan, 2003). Yet “findings of many studies remain unknown among designers and facility administrators” Day et al. (2000, p. 398).

Research on the impact of environmental modifications on neuropsychiatric symptoms and QoL in PwD has been extensively reviewed over the last two decades, providing theoretically and practically relevant knowledge for the academic world as well as for architects and other designers. To date a systematic, comprehensive review that includes evaluation of the strength of evidence is lacking. Reviews show that empirical research of the environmental design generally focuses on one or a few characteristics of the design to ensure validity, such as lighting (Guerry et al., 2020) or the support of wayfinding (Marquardt & Schmieg, 2009). Moreover, only some systematic reviews include evidence rating in their discussion (Anderiesen et al., 2014; Ferdous, 2020; Marquardt et al., 2014). This meta-review aims to build on consensual knowledge by reviewing existing reviews, thus presenting a comprehensive overview of specifications of the environmental design that are found to be associated with QoL and behavior, including assessment of the evidence level of these specifications. These results can be used to improve living environments for PwD.

## Method

### Search Strategy

In May and June 2022, a systematic review was performed on reviews that included combinations of the subjects environmental design, QoL, and behavior. The search strategy was developed with help of an information specialist and through discussion and consensus with second co-author; PRISMA-guidelines (Moher et al., 2009) were followed.

First, a preliminary search in literature published from 2009 through 2022 on these topics was conducted to establish the scope of the subject, keywords, and databases. This preliminary search yielded 16 journal articles, with 94 different keywords and 32 different databases. Second, the most frequently appearing keywords in the preliminary search were included in the search string, and the most frequently used databases were searched. These databases included CINAHL, Psychinfo, Medline, Embase, Pubmed, and Cochrane. Web of Science was added at a later stage to the databases for its broad range of content.

“physical environment” AND (dementia OR Alzheimer) AND (design OR “built environment”) AND review.

Only systematic reviews in English or Dutch were eligible; there was no restriction in time, nor were there any constraints on strength of evidence or study design. Reviews in the realm of home or community care, as well as reviews that focused on elderly in general instead of PwD, were excluded.

### Selection of Reviews

**Figure 1.**
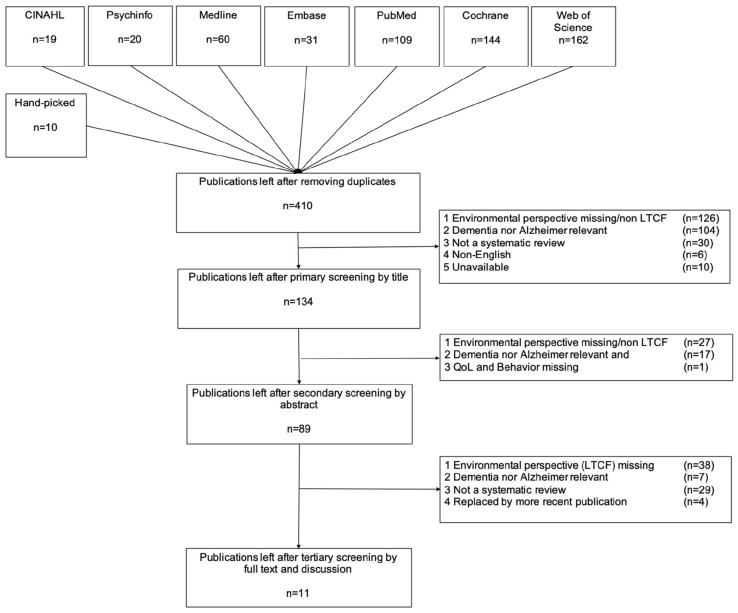
PRISMA flow diagram of the Literature review process.

The search string initially yielded 555 books and articles and 410 after eliminating duplicates, of which 276 items were discarded based on the title and 45 after analysis of abstracts. Ten articles were unavailable at the Dutch libraries. Of the remaining 88 articles, 78 were discarded based on irrelevance after full text-screening. Most of these items were removed because the title showed no relevance for the environment or dementia. The final result was 11 publications. A total of 250 underlying studies were retrieved to certify that the focus is on PwD, to eliminate multiple references in different reviews, and to access full text for analysis when necessary. Figure 1 shows the Prisma flow diagram of literature review process (Liberati et al., 2009).

### Strength of Evidence

In order to assess the quality of the extracted data, the level of evidence of underlying studies was summarized for each item. Of the 11 reviews, 8 contained some assessment of the strength of evidence of underlying studies by using different methods. No evidence rating was included in the remaining three reviews. The classification of the level of the evidence as described by Marquardt and Motzek (2013) was used (Table 1).

**Table 1.** Levels of Evidence for Healthcare Design.

Level	Description of quality
1	Systematic reviews of multiple randomized controlled trials (RCTs) or nonrandomized studies; meta-analysis of multiple experimental or quasi-experimental studies; meta-analysis of multiple qualitative studies leading to an integrative interpretation
2	Well-designed experimental (randomized) or quasi-experimental (nonrandomized) studies with a low attrition rate, intention to treat analysis, blinding, masked randomization, and consistent results compared to other, similar studies
3a	Observational studies with a cohort design, experimental, or quasi-experimental studies that did not fulfill the criteria of level 2
3b	Cross-sectional or case-control studies; qualitative research that, based on a literature review or a theoretical framework, reports a clear method and considers a diversity of views
4	Professional standards or guidelines with studies to report recommendations
5	Qualitative research that did not meet the criteria of level 3b
6	Recommendations from manufacturers or consultants who may have a financial interest or bias

The quality of the underlying studies varied and the majority of studies was based on moderate or weak study-designs or small numbers of data (level 3a or 3b).

### Data Extraction and Synthesis

The physical environment was described by the classification of features provided by Harris et al. (2002) (1) Ambient environment for example, lighting, noise, air quality, and odors, (2) Architectural features, for example, site, floorplan, size and shape of spaces, and placement of windows, (3) Interior design features, for example, furnishings and finishes, (4) Maintenance/Housekeeping, for example, cleanliness, wear, and clutter, and (5) Social features, for example, privacy, social engagement, wayfinding/orientation, and symbolic meaning for example, home likeness or institutional character. This classification is also used in the reviews by Woodbridge et al. (2018) and Anderiesen et al. (2014). QoL and behavior were labeled after Marquardt et al. (2014) (1) Behavior, for example, agitation, eating behavior, psychiatric symptoms, violence, and wandering, (2) Cognition, for example, attention and cognitive performance, (3) Function, for example, activities of daily living, falls, and mobility, (4) Well being for example, depressive symptoms, mood, and QoL, (5) Social abilities, for example, engagement and social interaction, (6) Orientation for example, wayfinding, and (7) Care Outcomes, for example, medication, oral intake, physical restraint use, and sleep. A standard data extraction form was used to record environmental specifications, aspects of QoL and/or behavior and evidence rating. Results are categorized by aspects of QoL and behavior according to Marquardt et al. (2014) and in addition, Supplemental Appendix 1 allows for digital categorization and selection. Supplemental Appendix 1 also allows for categorized according to design-principles as proposed by Fleming et al. (2017). Findings that show no or unclear association between environment and QoL or behavior were discarded. This selection was performed independently by two researchers. After discussion, consensus was reached on the final items.

## Results

Extracted data included 124 statements describing significant associations between specifications of the physical environment and QoL and/or behavior in PwD at evidence level 3a or higher (Supplemental Appendix 1).

### Behavior

#### Ambient Environment

The impact of ambient features like higher lighting levels and bright-light therapy (1,000–2,500 Lux) influenced residents’ agitation, and disruptive behavior and improved daytime wakefulness (Anderiesen et al., 2014; Chaudhury et al., 2018; Day et al., 2000; Fleming & Purandare, 2010; Marquardt et al., 2014) High noise-levels and increased cold-sensation perceptions were found to lead to agitation (Fleming & Purandare, 2010; Marquardt et al., 2014) Music, however, reduced agitation (Daly Lynn et al., 2019). In general, moderate or low levels of sensory stimulation were reported to prevent overstimulation, have a beneficial effect on agitation and reduce restraint use (Anderiesen et al., 2014; Day et al., 2000; Fleming & Purandare, 2010).

#### Architectural Features

LTCFs that are purpose-built for PwD, also called SCUs, offer segregated, specialized care by trained staff and a dementia friendly environment, typically in small-scale units. Such “dementia friendly environments,” although scarcely described, usually include a homelike ambiance and wayfinding support/signage (Ferdous, 2020). The reported impact on residents’ behavior of admission or relocation to an SCU are a reduction of behavioral disturbances and use of physical restraints (Fleming & Purandare, 2010) agitation and aggressive behavior (Soril et al., 2014), apathy and hallucinations, and an improvement of mobility (Day et al., 2000). Studies report that benefits like free access to and spending time in a garden or outdoor area included reduced agitation, aggression, drug use, and falls (Chaudhury et al., 2018; Soril et al., 2014; Whear et al., 2014; Woodbridge et al., 2018). Small unit-size (5–15 residents rather than 30) is associated with reduced agitation, aggression, and improved engagements in activities (Chaudhury et al., 2013; Day et al., 2000; Marquardt et al., 2014). However, evidence of the impact of unit-size is non conclusive. Studies reported by Marquardt et al. (2014) did not find any behavioral changes (Te Boekhorst et al., 2009) or worse, even more behavioral disturbances compared to traditional nursing homes (Kihlgren et al., 1992).

#### Interior Design Features

Homelike environments with open-plan dining areas and residential furnishings and finishes were reported to be associated with reduced verbal agitation and aggression, restlessness, trespassing, and exit-seeking (Chaudhury et al., 2017; Day et al., 2000; Fleming & Purandare, 2010).

#### Social Features

Personalized and non-institutionalized individual environments were found to be associated with a reduction of behavioral problems (Marquardt et al., 2014) such as agitation and aggression (Day et al., 2000). Contrarily again, some studies (Annerstedt, 1997; Elmståhl et al., 1997; Kihlgren et al., 1992) reported by Day found greater restlessness associated with a higher degree of homelikeness, due to greater assertion of independence (Day et al., 2000).

### Cognition

#### Ambient Environment

Finding a balance between overstimulation and sensory deprivation is one of the challenges in designing environments for PwD (Day et al., 2000; Marquardt et al., 2014) Moderate and low sensory stimulation were found to enhance residents’ concentration, thus possibly improving cognitive performance (Day et al., 2000).

#### Architectural Features

Evidence of environmental impact on cognition in PwD is limited. Some of the research mentioned by Annerstedt (1993, 1994, 1997) reviewed by Day et al. (2000) and Fleming and Purandare (2010) showed that small unit-size and group living may reduce intellectual deterioration. Ferdous (2020) stated that strong evidence supports positive outcomes of private bedrooms on neuro-disability, based on Calkins (2009).

### Function

#### Ambient Environment

Background music and exposure to higher overall light levels and all-day bright are reported to improve ADL-engagement and decrease functional decline (Anderiesen et al., 2014; Chaudhury et al., 2018; Fleming & Purandare, 2010).

#### Architectural Features

Residents of SCUs are reported to benefit from tailored architectural features by showing fewer declines in ADL-performance than in traditional environments (Boumans et al., 2019; Fleming & Purandare, 2010). More specifically, smaller unit-size of 5 to 15 residents and group living environments and green-care environments enhance involvement in activities and ADL-functioning (Chaudhury et al., 2018; Ferdous, 2020; Marquardt et al., 2014; Woodbridge et al., 2018). Literature is ambiguous about open-plan layouts. Although open-plan layouts are reportedly associated with more engagement in activities and better ADL-performance (Anderiesen et al., 2014; Day et al., 2000), Woodbridge concludes that enclosed rooms with clear functions are more supportive because they are easier to memorize. Providing a variation through a range of private and communal rooms (Woodbridge et al., 2018) and views to the garden were found to be positively correlated with activity.

#### Interior Design Features

General homelike environments were reported to enhance engagement in activities (Anderiesen et al., 2014), emotional and intellectual functioning, and autonomy, and reduce exit seeking (Chaudhury et al., 2018). Features of the dining area, a homelike décor and small group dining were found to contribute to the functional ability to take in food and fluids (Anderiesen et al., 2014; Chaudhury et al., 2018). Patterns and dark lines on flooring were found to be confusing and may cause falls (Marquardt et al., 2014).

### Well-being

#### Ambient Environment

Sensory stimulation must be controlled to evoke adverse effects (Marquardt et al., 2014); Lynn suggested removal of acoustic alarms leads to a calmer place (Daly Lynn et al., 2019). Controlled ambient sensory stimulation for example, background music or singing may were found to improve residents sense of vitality (Anderiesen et al., 2014), and multisensory environments, the so-called “snoezelen” in Dutch, had a positive effect on mood (Marquardt et al., 2014; Soril et al., 2014). Finally, one effect of all-day exposure to higher light-levels (2,500–10,000 Lx) is improved mood (Chaudhury et al., 2018; Fleming & Purandare, 2010).

#### Architectural Features

Living in tailored environments like an SCU, small unit-size, group living and a familiar, non-institutionalized design and homelike environment were found to be beneficiary for residents’ well-being (Boumans et al., 2019; Chaudhury et al., 2018; Day et al., 2000; Ferdous, 2020; Marquardt et al., 2014). Again, preventing uniformity by varying ambiance of rooms were found to reduce depression and hallucinations (Fleming & Purandare, 2010). Green care farms, including the presence of animals and gardens provide opportunities for attractive outdoor activities and were reported to be associated with improved psychological well-being (Chaudhury et al., 2018; Ferdous, 2020; Whear et al., 2014). In addition, family caregivers experienced less burden in small-scale living facilities (Boumans et al., 2019; Fleming & Purandare, 2010), and perceived improved QoL in residents living in an SCU (Fleming & Purandare, 2010). Technology assists, for example, for opening a bedroom door may be supportive for resident’s sense of autonomy (Boumans et al., 2019) and an integrated camera-circuit were reported to be associated with reduced privacy invasion (Daly Lynn et al., 2019).

#### Social Features

In addition to remarks on homelike environments above, improved well-being was reported by support of orientation and wayfinding, for example, by the use of colors (Anderiesen et al., 2014), or personalization (Marquardt et al., 2014).

### Social Abilities

#### Architectural Features

Living in specialized, small-scale units was reported to improve communication skills, social interaction and improve relationship between residents and formal caregivers (Boumans et al., 2019; Day et al., 2000).

#### Social Features

Several authors pointed out that homelike and noninstitutional residential environments enhance social interaction and communication (Anderiesen et al., 2014; Chaudhury et al., 2018; Marquardt et al., 2014; Woodbridge et al., 2018). Dining “family-style” in small groups increased social interaction; low social density in general was positively associated with social abilities; residents in small groups were found to be more engaged in each other and have fewer conflicts (Marquardt et al., 2014) . Providing variation in private and public spaces facilitated different kinds of communication (Woodbridge et al., 2018). In reducing loneliness, social robot “Paro” is found to be effective (Daly Lynn et al., 2019).

### Orientation

#### Ambient Environment

Higher light levels and exposure to daylight improved orientation (Day et al., 2000; Fleming & Purandare, 2010).

#### Architectural Features

Legibility of the architectural environment was found to support spatial orientation; this can also be improved by simple layouts with two-way decision pathways (Woodbridge et al., 2018), visibility of relevant spaces (Marquardt et al., 2014) and layouts that are H or L-shaped (Chaudhury et al., 2018; Ferdous, 2020). Long hallways were found to impede residents’ orientation (Chaudhury et al., 2018), but spaciousness of hallways supports orientation (Chaudhury et al., 2018). Van Steenwinkel describes the concept of spatial articulation, the configuration of distinctive private, less private to public layers in the home environment that allow residents to adjust to the environment step by step (Van Steenwinkel et al., 2012). Well-articulated spaces with distinctive functions contribute by their symbolic meaning to this process of adjustment from private to public environments and orientation. Environmental cues to support this were size, proportion, materiality and furnishings, personalization, use of color, texture, signage containing icons and text, and use of landmarks at decision points (Ferdous, 2020; Marquardt et al., 2014; Woodbridge et al., 2018).

#### Interior Design Features

Personalization through nameplates, portrait-type photographs, use of texture and colors enhanced resident’s ability to find his or her own room (Marquardt et al., 2014; Woodbridge et al., 2018). Combinations of color and material that give meaning to spaces or functions were found to help residents’ orientation (Guerry et al., 2020). In a broader perspective, preventing uniformity by providing different zones with a unique character enhances wayfinding abilities.

#### Social Features

Orientation cues and wayfinding aids, including the use of color, landmarks, and signage may improve orientation (Ferdous, 2020; Woodbridge et al., 2018).

### Care Outcomes

#### Ambient Environment

Spaces with low-sensory stimulation were found to play a role in reducing weight loss (Fleming & Purandare, 2010) and exposure to bright light during the day improved the circadian rhythm and quality of sleep (Anderiesen et al., 2014; Chaudhury et al., 2018; Marquardt et al., 2014).

#### Architectural Features

Several features of architectural design were found to be associated with improved care outcomes, for example, drug use and quality of sleep. First, small size group living environments and SCUs reduced psychotropic drug-use (Day et al., 2000; Fleming & Purandare, 2010; Marquardt et al., 2014). Second, a low social density improved general care outcomes (Marquardt et al., 2014). Third, the use of outdoor and garden areas and participation in outdoor activities reduced drug-use and improve quality of sleep (Chaudhury et al., 2018; Whear et al., 2014).

#### Interior Design and Symbolic

features Several authors (Chaudhury et al., 2018; Day et al., 2000; Woodbridge et al., 2018) mention that a homelike, family-style dinner interior was related to increased food intake, as well as introducing an aquarium into dining settings (Woodbridge et al., 2018). Food and fluid intake was also found to improve using high-contrast tableware (Chaudhury et al., 2018; Woodbridge et al., 2018).

## Discussion

The results of this review show that a broad range of features of the physical environment are beneficiary to QoL and behavior in PwD based on a large body of literature. A total of 124 items met the required evidence-levels 1–3a (Supplemental Appendix 1). However, the general evidence level is not strong.

Small-scale and specialized care units are found to positively impact behavior, well-being, communication skills, engagement in activities, ADL-functioning, intellectual performance, orientation, food-intake, and decrease psychotropic drug use. The use of outdoor and garden areas and participation in outdoor activities are found to reduce psychotropic drug-use and improve quality of sleep. Homelike interior environments are associated with reduced behavioral disturbances, improved well-being, social interaction, and engagement in activities. Family-style dining in small groups enhances social interaction and food and fluid intake. Higher light levels and exposure to bright light during the day improves engagement in activities and reduces functional decline, while improving the circadian rhythm and quality of sleep.

Finding a balance between overstimulation of senses and sensory deprivation is a first challenge in designing environments for PwD. In general, moderate to low levels of sensory stimulation for example, background music or singing, prevent overstimulation. Several authors (Ferdous, 2020; Fleming & Purandare, 2010; Woodbridge et al., 2018) have observed that providing a variation through a range of distinctive and well-articulated private and communal rooms with garden views and measures to prevent uniformity by varying ambiance of spaces have beneficiary effects on behavior and the QoL. This relates to the concept of spatial articulation (Van Steenwinkel et al., 2012), the configuration of distinctive private, less private to public layers in the home environment, which is found to support orientation and way-finding.

The provision of varying environments and ambiances that offer choices to residents in varying levels of privacy, sensory stimulation, and distinctive functions, requires a diversity of available spaces and may therefore be at odds with the concept of family-like small-scale facilities. This point has also been stressed by Van Steenwinkel et al. (2017) who challenges family-like group living and underlines the importance of freedom of activity and choice. Variety and freedom of movement allow for that choice. This dualism poses a second design challenge for architects, interior decorators, and landscape gardeners; guidelines like TESS-NH (Sloane et al., 2003) ignore this dualism currently and need to be reevaluated on this point.

### Strengths and Limitations

The review process has been comprehensive and systematical, and a large body of literature was reviewed, including an assessment of the level of evidence. Although the level of this evidence is limited, the congruence of different studies and reviews gives strength to the outcomes of this review. However, a review of reviews is vulnerable to differences in used methodology, search engines used, methods of evidence rating and methods of reporting. We addressed this by focusing on the Results sections of the reviews. Nevertheless, results may still be at risk of bias of positive reporting, since only significant associations between environment and QoL and behavior have been included and findings that show no or unclear associations were discarded. Evidence rating was based on the robust classification of research design by (Marquardt and Motzek, 2013) and already used in Marquardts extensive review (Marquardt et al., 2014) As a consequence, extra information of more detailed measures for evidence rating used by other authors, for example, Fleming and Purandare (2010) was lost by this method. In case a review included evidence rating or description of used methods, the classification of the author was followed. For reviews that lacked evidence rating, the classification was performed independently by two researchers (AS, SJ; Boumans et al., 2019; Chaudhury et al., 2018; Daly Lynn et al., 2019; Woodbridge et al., 2018). Studies that are included in multiple reviews cause a risk of bias. This risk was eliminated by merging multiple references to one underlying study. Further elimination of bias, for example, by selection of articles, was prevented by including only systematic reviews.

In addition, the average year of publication of the included reviews is slightly above 2014, against an average of 2006 for the underlying studies. Although this timeframe is an inherent disadvantage of this type of study, it raises the question whether the results may have become obsolete. Since then, care dependency of PwD in LTCFs had increased significantly (Ministerie van Volksgezondheid Welzijn en Sport, 2022). This may be relevant because the impact of the physical environment on QoL and behavior may become different as dementia progresses.

## Conclusion and Recommendations

The main goal of this review is to present a comprehensive overview of specifications of the environmental design that are claimed to be associated with quality of life and behavior, including evidence level. The results show broad opportunities to improve environmental design that support quality of life and behavior. This review has produced a comprehensive overview of characteristics of architectural and interior design and links to behavior, cognition, function, well-being, social abilities, orientation, and care outcomes and may be used as a tool for interventions.

Even though more empirical research is needed to collect higher-level evidence in general to assess the impact of various interventions in the physical environment, we can state that finding a balance between overstimulation of senses and sensory deprivation is a challenge in designing LTCFs. Also, making a range of distinctive varying environments and ambiances available in order to facilitate freedom of movement and choice, simultaneously ascertaining a homelike atmosphere and small-scale environments, poses a challenge throughout the entire design process.

We recommend using the results in new developments and refurbishments of LTCF’s as a design guideline. Because the included reviews in this study nor the underlying studies have yielded guidelines for the nature of varying ambiances of different spaces, this needs further research. Architects as well as landscape, interior, and other designers are challenged to contribute to a supportive physical environment by designing appropriate, small-scale, homelike but varying spaces to meet the needs of the residents.

## Supplemental Material

sj-xlsx-1-ggm-10.1177_30495334251345092 – Supplemental material for The Physical Environment and the Quality of Life and Behavior in People With Dementia: A Systematic Meta-Review Supplemental material, sj-xlsx-1-ggm-10.1177_30495334251345092 for The Physical Environment and the Quality of Life and Behavior in People With Dementia: A Systematic Meta-Review by Arnout Siegelaar, Mark P. Mobach, Sarah Janus and Sytse U. Zuidema in Gerontology and Geriatric Medicine
